# Prognostic factors and outcome of Post-Neonatal Tetanus in an intensive care unit of a Tertiary Care Hospital

**DOI:** 10.12669/pjms.35.5.656

**Published:** 2019

**Authors:** Nighat Sultana, Attia Bari, Mehwish Faizan, Muhammad Sarwar

**Affiliations:** 1 Dr. Nighat Sultana, MCPS, FCPS. Department of Pediatric ICU, The Children’s Hospital and The ICH, Lahore, Pakistan; 2 Dr. Attia Bari, DCH, MCPS, FCPS, MHPE. Department of Pediatric, The Children’s Hospital and The ICH, Lahore, Pakistan; 3 Dr. Mehwish Faizan, FCPS. Department of Pediatric, The Children’s Hospital and The ICH, Lahore, Pakistan; 4 Dr. Muhammad Sarwar, MCPS, FCPS. Department of Pediatric ICU, The Children’s Hospital and The ICH, Lahore, Pakistan

**Keywords:** Tetanus, Autonomic instability, Children, Neurological, Outcome, Ventilation

## Abstract

**Objective:**

To determine the prognostic factors and outcome of tetanus in children of post-neonatal age admitted in the intensive care unit (ICU) of a tertiary care hospital.

**Methods:**

This prospective cross sectional study, carried out in the Pediatric ICU of The Children’s Hospital Lahore from Jan 2013 to March 2017. Children of both genders with age range of two months to 16 years diagnosed clinically as tetanus were included. All 132 patients were scrutinized for all possible risk factors, need for mechanical ventilation and outcome. Data was analyzed by SPSS version 20.

**Results:**

Mean age of children was 7.5±3.4 years with male predominance (70.5%). Only (38.6%) received three doses of vaccination but none had booster dose. Trauma (43.2%) encompassed maximum predisposing factor followed by ear or nose prick and ear discharge. Mean duration of ICU stay was 20±13.3 days. Mortality rate was (17.4%). Ventilator support was given to (78.8%). Neurological outcome was normal in (82.6%). Trauma, ear or nose prick in girls and ear discharge were significantly associated with poor outcome and death with p-value of <0.001, 0.011 and <0.001 respectively. Other factors associated with poor outcome were need for mechanical ventilation and neurological impairment with p-value of 0.001 and <0.001 respectively.

**Conclusion:**

Tetanus is causing our children to suffer from devastating disease. Vaccination status is not satisfactory and along with trauma, ear discharge and ear or nose prick are identifiable risk factors. To combat these issues large scale vaccination and booster doses remains promising option.

## INTRODUCTION

Tetanus is a vaccine preventable disease but even in this era of effective vaccines against most preventable diseases tetanus is still considered a common public health problem particularly in developing countries causing huge morbidity and mortality. However, in resource rich countries with the help of a good immunization coverage the disease incidence is decreased with very low mortality rates.^1^

Tetanus is caused by a potent exotoxin called tetanospasmin produced by gram positive organism Clostridium tetani.^2^ Tetanus is the only vaccine-preventable disease which is not communicable and is acquired through environmental exposure to the spores of Clostridium tetani.^3^ It is an acute neuromuscular disorder diagnosed clinically and is characterized by increased muscle tone, rigidity and spasms caused by neurotoxin. There may be autonomic dysfunction and in severe forms patients with tetanus requires respiratory and hemodynamic support.^4^

In most cases of tetanus an acute penetrating injury is the preceding event but sometimes the injury is so mild and it often go unnoticed without seeking medical treatment. The other factors which may be associated with tetanus are chronic skin ulcers, burns, surgery, abscesses, gangrene, ear discharge and ear or nose pricks using unhygienic needles. The portal of entry of clostridium tetani is not identifiable in all cases.^5^

The main reason for developing this deadly disease is low immunization coverage and deficient booster doses of tetanus toxoid at appropriate period in the immunization schedule contributing to the high disease burden in developing countries. There are many other factors involved in this disease including lifestyle, social influence and living conditions particularly in rural areas and in warm climates.^6^

In our experience, substantial number of tetanus cases are admitted with severe disease. There are number of studies regarding risk factors available with very few studies from Pakistan particularly documenting admission and monitoring in Intensive care unit (ICU) setting and outcome of children with severe tetanus. There is a need and room to document our experience about this aspect. Hence, we planned this study to analyze clinical characteristics, prognostic factors and outcome of post-neonatal tetanus cases admitted to ICU of our tertiary care center.

## METHODS

This was a prospective cross sectional study, carried out in Pediatric ICU of The Children’s Hospital & The Institute of Child Health (ICH), from Jan 2013 to March 2017 after the approval from Institutional Review Board. We used a Questionnaire Performa for data collection and before data collection informed written consent from parents was taken. As the exact prevalence of post neonatal tetanus in the developing countries is not known so the sample size was calculated to be 132 by taking 8-15% deaths in children due to tetanus. Non-probability convenience sampling method was used. Children of both gender with age range of two months to 16 years who were diagnosed as tetanus on the clinical basis including tetanic spasms, locked jaw and clear conscious level were included. While patients less than two months and having seizures due to any other disease or past history of neurological disease were excluded from the study.

In all included patients age, sex, duration of illness, presenting symptoms were documented and all 132 patients were scrutinized for possible risk factors, need for mechanical ventilation, post ventilation neurological status and outcome in the form of discharged, died, left against medical advice (LAMA) and transferred out of ICU to a medical ward after stabilization and discharged.

### Statistical analysis

Data was analyzed by SPSS version 20. Descriptive statistics were used to describe the demographic details as mean and percentages. Statistical analysis was performed using chi-square test to see association between outcome and age group, duration of illness and ventilation requirement. A p-value of less than 0.05 was considered significant.

## RESULTS

The results of our study showed predominance of males 93 (70.5%) over females 39 (29.5%). Mean age of children was 7.5 ± 3.4 years. Major proportion of patients were in the age range of 5.1-10 years constituting 60 (45.5%) of total patient, while 40 (30.3%) cases were in age group less than 5 years. Majority belonged to urban areas 101 (70.5%). Only 51 (38.6%) received three doses of tetanus toxoid but none had any booster dose (Table-I).

**Table I:** Demographics of study population.

Characteristics	Frequency (n)	Percentage
***Age***		
1-5 Years	40	30.3
5.1-10 Years	60	45.5
10.1-14 Years	32	24.2
***Sex***		
Male	93	70.5
female	39	29.5
***Address***		
Rural	101	76.5
Urban	31	23.5
***Vaccination status***		
1 dose	38	28.8
2 doses	43	32.6
3 doses	51	38.6
Booster dose	0	0

**Fig.1:**
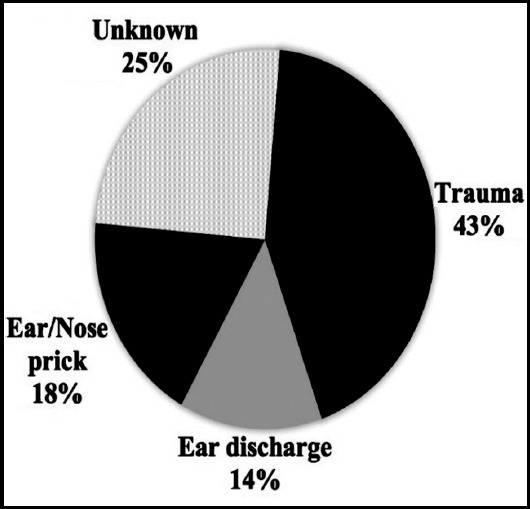
Portal of entry in post neonatal tetanus.

Definite history of trauma was positive in 57 (43.2%) patients. Other predisposing factors shown in (Fig.1).

Mean duration of symptoms was 8.3±3.2 days. Mean duration of ICU stay was 20±13.3 days. Out of 132 patients 104 (78.8%) patients required mechanical ventilation due to intractable seizures and respiratory compromise. In these 104 patients 25(19%) who had difficulty in weaning from ventilation required tracheostomy. Mean duration of mechanical ventilation was 18±10.5 days. A significant proportion had autonomic instability 75(56.8%). Nosocomial infection occurred in 97(73.5%). Majority had normal neurological outcome 109(82.6%). In our study mortality rate was 17.4%, maximally involving children 5.1-10 years (20%). Seventeen (12.9%) patients left against medical advice due to critical condition (Table-II).

**Table II:** Factors and Outcome of patients with Tetanus during ICU Stay.

Factors & Outcome	Frequency (n)	Percentage (%)
Need for Mechanical Ventilation	104	79
Tracheostomy	25	19
MgSo_4_	25	19
Autonomic instability	75	57
Nosocomial infection	97	74
Normal neurological outcome	109	83
***Outcome***		
Discharged	16	13.6
Died	23	17.4
LAMA	17	12.9
Shifted to medical ward after stabilization	74	56.1

Trauma, ear or nose prick in girls and ear discharge were significantly associated with poor outcome and death with p-value of <0.001, 0.012 and <0.001 respectively while we could not establish significant association of nosocomial infection with outcome p-value 0.053. Other factors associated with poor outcome were patients with intractable seizures requiring MgSO_4_, need for mechanical ventilation and neurological impairment with prolonged ventilation and brain anoxia with p-value of 0.003, 0.001 and <0.001 respectively (Table-III).

**Table III:** Factors affecting outcome of patient with Tetanus in intensive care unit.

Variables	Outcome				p-value

	Discharged	Died	LAMA	Transfer out	
MgSO_4_ given	5(19.2%)	7(26.9%)	6(23.1%)	8(30.8%)	0.033
Mechanical ventilation	16(15.4%)	22(21.2%)	17(16.3%)	49(47.1%)	0.001
***Neurological outcome***					
Normal	18(16.5%)	11(10.1%)	10(9.2%)	70(64.2%)	<0.001
Impaired	0(0%)	12(52.2%)	7(30.4%)	4(17.4%)	

## DISCUSSION

Tetanus is a serious illness and often a life-threatening disease. The relative incidence of tetanus is higher in neonatal age group due to improper sterilization but tetanus is still seen in older age group in under developed countries with inadequate vaccination coverage. In our study the children were in post neonatal age and we found that mean age of presentation was 7.5±3.4 years consistent with 8 years in a study from Ethiopia and a study by Alhaji et al. showing mean age of 6.9±3.87 years.^2^,^7^ Our study revealed that brunt of burden is born by age group of 5-10 years with 45.5% cases followed by <5 years (30.3%). This pattern of age distribution is being endorsed by many other researchers.^1^,^7^,^8^ A study done by Oyedeji et al. showed tetanus in a slightly older age group as the highest prevalence was seen in age group of 10-15 years (54.5%), followed by 22.7% each >1 month to 5 years and >5 to 10 years.^9^ In majority of researches on tetanus from all over the world there is male preponderance. We had 70% of male children affected by tetanus and a similar Male: Female ratio of 1.7: 1.0 is described in a research from Nigeria.^10^ A male predominance confirmed in our study is consistent with findings of many other researchers showing that male preponderance is due to the fact that male children are more prone to accidental injuries due to their adventurous nature which puts them at greater risk of injuries.^9^,^11^

As far as the portal of entry is concerned trauma is identified as the major cause in many studies and regarding the site of trauma, majority of the children had injury to lower limbs or foot. In our study, there was significant history of trauma in 43% of patients but still in almost 25% no portal of entry was identified. Other causes were ear and nose pricks by unhygienic techniques (18%) and ear discharge in 14%. Similar findings are shown in a study by Junejo et al. documenting wound as a source of infection in 60.8%, discharging ear 21.62%, and cause remained unidentified in 26 17.56%.^8^ Ear infection is young children is often taken lightly but almost 33.3% of tetanus occurred through this route documented in a study by Alhaji et al.^7^ A study from India showed ear infection as the most common route of infection 49% followed by trauma 29%.^12^

Despite enormous efforts of our health care authorities and our government the vaccination status of Pakistani children in general is not good. We found that only one third of children who presented with tetanus had received three doses of tetanus toxoid but none of the children received booster dose. A study from Pakistan showed much worse results about the vaccination status showing that none of patients was vaccinated for tetanus.^13^ Majority of our children were not immunized or partially immunized, which is similar to reports by other workers and a study by Oyedeji et al. showed exactly similar ratio of completely vaccination and partially or totally unvaccinated children.^1^,^5^,^9^ Another factor responsible for these tetanus cases is that routine EPI schedule doesn’t contain any booster dose after one year of age and there is no awareness of adult immunization in Pakistan. Regarding the lack of awareness and practices about the booster doses a study from Nigeria teaching hospital showed that none of the patients received booster dose after their first birthday.^9^ Quite contradictory to this fact of lack of booster doses of tetanus toxoid a study from a developing country by Tadele showed booster vaccination in 29% of the patients.^2^

Prolonged hospitalization averaging 3-4 weeks reported by various workers is in agreement with the three weeks amongst our patients. Mean duration of ICU stay in our study was 20±13.3 days consistent with the finding of a study done by Animasahun in which average length of stay was 20 days.^10^ Prolonged length of stay in ICU of 2.5-7 weeks were for mechanical ventilator support, tracheostomy requirement for respiratory compromise or antispasmodic medications such as magnesium sulphate.^14^

While managing patients with tetanus, autonomic instability, nosocomial sepsis and ventilator-associated pneumonia are major issues faced in the intensive care setting. We faced autonomic instability in 56% of our patients and nosocomial infection occurred in 73.5%. Magnesium sulphate has been used both in artificially ventilated patients to reduce autonomic disturbance and in non-ventilated patients to control spasms. Few studies also mentioned these two common problems in tetanus patients managed in ICU setting.^4^,^15^ In our study one third of total patients required mechanical ventilator support and 19% patients had tracheostomy. Neurological impairment although not a feature of tetanus but we encounter it in 17% of patients requiring prolonged ventilation and resulting from brain anoxia. In a study published by Muazzam et al. 41% patients required tracheostomy, 39.9% patients did not require any respiratory support and endotracheal tube was needed in 5%.^5^ Case fatality rate in our study was 17.4% consistent with a study Oyedeji showing mortality rate of 18%. A study from nigeria showed the lowest mortality of 5.9%.^16^ A much higher fatality rate was observed in various researches ranging from 26% to 39% and a study from Nigeria showed case fatality rate of 61% in post neonatal tetanus.^17^

### Strengths and Limitations

Our strength is a large sample size as most studies from all around the world had a much smaller sample. Limitation is that we did not collect the data regarding parental education level which has a significant impact on the vaccination status of the child.

## CONCLUSION

Post neonatal tetanus has remained a major public health problem with high morbidity and mortality rates especially in developing countries like ours. Tetanus is causing our children to suffer from devastating disease. Vaccination status is not satisfactory and booster doses are not taken into account. Along with trauma, ear discharge and ear or nose prick are identifiable risk factors. Ear discharge in pediatric population is an ignored entity and special attention should be given to discharging ears. To combat these issues large scale vaccination and booster doses remains promising option and health education regarding care of the wound and ear discharge is also needed.

### Author`s Contribution

**NS**: Conceived, designed, data collection.

**AB**: Statistical analysis and manuscript writing.

**MF:** Review, suggestions.

**MS:** ICU patient monitoring.
